# ADAR1 is vital for B cell lineage development in the mouse bone marrow

**DOI:** 10.18632/oncotarget.11029

**Published:** 2016-08-02

**Authors:** Victoria Marcu-Malina, Sanja Goldberg, Einav Vax, Ninette Amariglio, Itamar Goldstein, Gideon Rechavi

**Affiliations:** ^1^ Sheba Cancer Research Center, Chaim Sheba Academic Medical Center, Tel Hashomer, Israel; ^2^ Department of Pediatric Hemato-Oncology, Chaim Sheba Academic Medical Center, Tel Hashomer, Israel; ^3^ Rheumatic Diseases Unit, Chaim Sheba Academic Medical Center, Tel Hashomer, Israel; ^4^ Sackler School of Medicine, Tel-Aviv University, Tel-Aviv, Israel; ^5^ The Mina and Everard Goodman Faculty of Life Sciences, Bar Ilan University, Ramat Gan, Israel

**Keywords:** RNA editing, ADAR1, B cell, lymphopoiesis, epitranscriptomics, Immunology and Microbiology Section, Immune response, Immunity

## Abstract

Adenosine deaminase acting on RNA (ADAR) 1 is the master editor of the transcriptome, catalyzing the conversion of adenosine to inosine (A-to-I). RNA transcripts fold into a variety of secondary structures including long intramolecular RNA duplexes that are the major substrate of ADAR1. Most A-to-I editing sites occur within RNA duplexes formed by complementary pairing of inverted retrotransposable elements interspersed within noncoding regions of transcripts. This catalytic activity of ADAR1 most likely prevents the abnormal activation of cytosolic nucleic acid sensors by self-dsRNAs. Homozygous disruption of mouse *Adar* is embryonic lethal due to a toxic type-I interferons response and correspondingly biallelic missense mutations in human *ADAR1* cause a severe congenital interferonopathy. Here, we report that Cd19-Cre-mediated *Adar* gene ablation in the mouse causes a significant defect in the final stages of B cell development with an almost complete absence of newly formed immature and CD23^+^ mature recirculating B cells in the BM. *Adar* ablation in pre-B cells induced upregulation of typical interferon-stimulated genes (ISGs) and apoptosis upon further maturation. ADAR1 deficiency also inhibited the *in vitro*, IL-7-mediated, differentiation of BM-derived B cell precursors. In summary, ADAR1 is required, non-redundantly, for normal B lymphopoiesis in the BM and peripheral maintenance.

## INTRODUCTION

RNA molecules undergo elaborate post-transcriptional modification, such as editing and methylation, collectively termed epitranscriptomics [1–3]. The most prevalent type of RNA editing in mammalians is the deamination of adenosine into inosine (A-to-I editing) primarily catalyzed by ADAR1. The *Adar* gene encodes a constitutively expressed p110 isoform and a longer interferon-inducible p150 isoform. The two isoforms have different translation initiation sites and promotors. The p110 isoform is present predominantly in the nucleus whereas the interferon-inducible p150 isoform is usually cytoplasmic [4]. The development of bioinformatics methodologies and tools to map A-to-I editing sites revealed that they are highly abundant in untranslated regions (UTRs) of mRNAs [1, 5]. The majority of editing within UTRs is promiscuous, occurring within long intramolecular RNA duplexes formed by complementary pairing of inverted repeats: e.g., *Alu* retrotransposons in the human and orthologous short interspersed repetitive elements (SINE) in the mouse [1, 6]. For example, the human transcriptome contains dense clusters of A-to-I editing sites, termed as hyper-edited, localizing mostly in *Alu* repeats [7].

Genetic ablation of *Adar* in the mouse is embryonic lethal at midgestation due to defective hematopoiesis and a detrimental type-I interferons response [8–12]. Similarly, infants with mutated *ADAR1* develop during early life a severe interferonopathy, the Aicardi-Goutières syndrome (AGS), presenting with encephalopathy and features akin with systemic lupus erythematosus (SLE) and congenital viral infections [13–16]. Recent studies in mouse models show that deletion of genes involved in the innate immune response to double-stranded (ds)RNA rescues ADAR1 deficient mice to birth [8, 17, 18]. Taken as a whole these observations imply that a major biological function of *Adar* is hyperediting of repetitive elements in the transcriptome to prevent abnormal stimulation of nucleic acid sensing pathways [19, 20]. ADAR1 has been shown to have other conserved biological functions, as follows: (i) infrequent but important modifications of codons within mature mRNAs [21]; (ii) editing-dependent modifications of the alternative splicing of selected pre-mRNAs [22]; (iii) editing-independent and dependent modulation of microRNA biogenesis and/or function [23, 24].

Despite the fact that ADAR1 is essential for embryonal development, normal fetal and adult hematopoiesis, and maintaining innate immune homeostasis in the mouse and human [19, 20], no previous studies have addressed its role in B cell development. In this study, using *in vivo* Cd19-Cre-mediated conditional ablation of *Adar* gene, we asked how ADAR1 deficiency affects the late stages of B lymphopoiesis predominantly in the BM.

## RESULTS AND DISCUSSION

### CD19-Cre^ki^-mediated conditional ablation of Adar inhibits B lymphopoiesis

ADAR1 is required for the early stages of fetal and adult hematopoiesis [10, 25]. We now asked whether ADAR1 is also vital for the late phases (from pre-B cell stage and onward) of adult B lymphopoiesis, using a model system of Cd19-Cre^ki^-mediated recombination of floxed *Adar*. The B cell lineage develops initially in the BM from common lymphoid progenitors (CLP) through sequential stages: developing from CLP to pre-pro-B cells (Hardy classification Fr. A), to pro-B cells (Fr. B/C), to pre-B cells (Fr. D), and lastly to immature/new B cells (Fr. E) [26]. The expression of the CD19 molecule is a marker for B lineage-committed cells, and normally its expression starts at the pro-B cell stage [27]. We selected B cell-specific expression of Cre from the *Cd19* locus over expression from the *mb1* gene, as the efficiency of target gene recombination in the first model system is much lower in early precursors (Fr. B/C), increasing significantly as the B cell mature reaching efficiency of 80-95% in Fr. E to F cells [28]. We used two relevant mouse genotypes Adar^fl/fl^Cd19-Cre^ki/+^ and Adar^fl/fl^Cd19^ki/ki^ to address our question, predicting that the second mutant, homozygous for the Cd19-Cre^ki^ allele, would display a higher penetrance of biallelic ablation of the floxed *Adar* gene segment in late B cell precursors.

**Figure 1:**
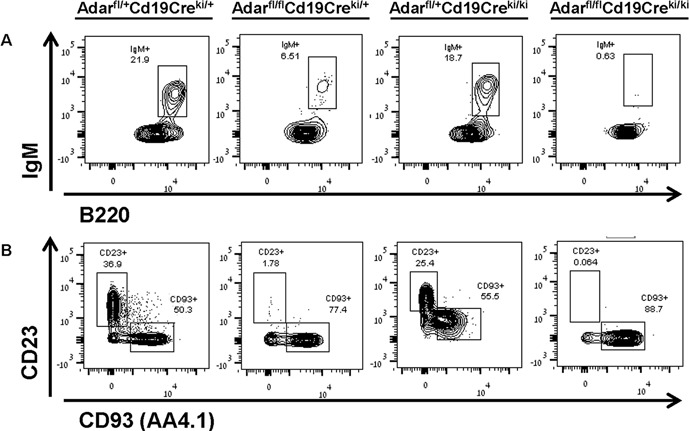
Cd19-Cre-mediated *Adar* disruption causes profound deficiency of immature and recirculating mature B cells in the BM Single cell suspension of BM cells were obtained from 8-16 week-old mice and depleted of erythrocytes. Next, to identify B cell precursors in the last stages of B-lymphopoiesis we immunostained the samples with flourochrome-conjugated mAbs as detailed in Materials and Methods. Samples were acquired by a FACSAria instrument and the data (> 10,000 events per sample) were analyzed/plotted using the FlowJo V.10 software. **A.** Upper contour plots depict the percentage of immature IgM^+^ B cells in the indicate group (21.9. 6.51, 18.7 and 0.63, respectively). **B.** Lower plots show the percentage of MR CD23^+^CD93^−^ B cells in the indicate group (36.9, 1.78, 25.4, and 0.064; respectively) and other less mature B-lineage cells (50.3, 77.4, 55.5, and 88.7; respectively). The data shown are from a representative mouse from each of the four indicated genotypes.

**Figure 2:**
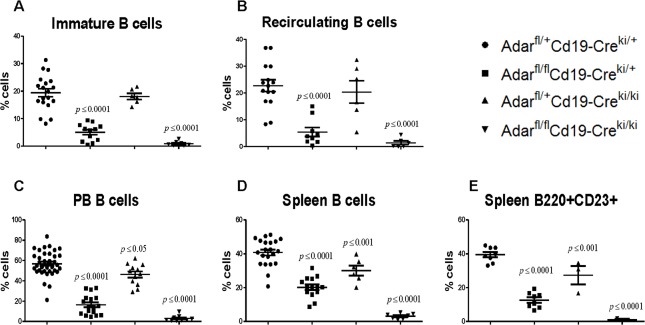
B lineage specific *Adar* ablation results in a profound deficiency of immature (naïve) and peripheral (mature) B cell deficiency Charts show the percentage of **A.** immature and **B.** MR B cells in the BMs from a large number of mutants and littermate control mice. **C.** Percentage of peripheral blood circulating B cells in the four indicated mice groups. Percentage of **D.** total B220^+^ and **E.** transitional T2/follicular CD23^+^ splenic B cells. Each point in the graphs represents the percentage of the specified B cell subset for an individual mouse. Mean values are represented with horizontal bars, and error bars represent ±SD. *P* values were determined by the one-way ANOVA test (compared against Adar^fl/+^Cd19-Cre^ki/+^ controls).

The FACS analysis of freshly isolated BM cells from Adar^fl/fl^Cd19-Cre^ki/+^ mice for the major stages of B lymphopoiesis in the BM, revealed a significant reduction of ~70% in the number of immature B cells (Fr. E) compared to littermate Adar^fl/+^Cd19-Cre^ki/+^ control mice (Figure 1A and combined data in Figure 2A). In addition, these mutants also showed a very significant reduction of ~75% in mature recirculating (MR; Fr. F) CD23^+^CD93^−^ B cells (Figures 1B and combined data in Figure 2B). As predicted, the Adar^fl/fl^Cd19-Cre^ki/ki^ mice displayed a more profound, less variable, reduction of ~95% in the percentage of immature B cells compared to littermate controls, and consequently an almost complete lack of MR B cells (Figures 1A-1B, and combined data in Figures 2A-2B). However, these two mutant mice groups did not show a significant reduction in the development and maintenance of more premature B cells precursors, exhibiting normal numbers of Fr. B to D cells in their BMs (data not shown). The percentages of CD3^+^ T cells and monocytes in their BM were also normal (data not presented).

Newly formed conventional B cells (Fr. E) undergo further maturation in the periphery. Therefore, we also determined the effect of *Adar* ablation on mature splenic and peripheral blood (PB) circulating B cells. The combined FACS data showed, yet again, a significant reduction in the percentage of PB circulating B cells, total splenic B cells, and transitional T2 plus mature CD23^+^ splenic B cells in the two mutant mice groups compared to respective littermate controls. As expected, the block in B cell peripheral maturation and maintenance was more substantial in Adar^fl/fl^Cd19-Cre^ki/ki^ compared to Adar^fl/fl^Cd19^ki/+^ mutants. The findings that the deleterious biological consequences of Cd19-Cre^ki^-mediate *Adar* gene ablation manifested on or after the immature B cell stage (Fr. E to F) agree with our prediction, based on previous data [28], that the penetrance of Cd19-Cre-mediated *Adar* recombination should become extensive only in the late phases of B lymphopoiesis.

Previous data from the Rajewsky laboratory on the phenotype of homozygous Cd19-Cre^ki^ mice, lacking surface CD19 expression, show that conventional B cell development in their BM was undisturbed. However, their peripheral B cells showed a highly reduced capacity to respond to T cell-dependent antigens and for antibody affinity maturation [27]. Moreover, while CD23^+^ transitional T2 and follicular splenic B cells were present in adequate numbers in CD19 deficient mice, the subset of innate-like CD23^−^ marginal zone B cells was practically absent [29]. We also found grossly normal percentages of Fr. B to E cells in the BM of Adar^fl/+^Cd19-Cre^ki/ki^ mice and only a minimal reduction in the percentage of CD23^+^ splenic B cells (Figures 1–2 and unpresented data). Taken as a whole these observations lead us to conclude: (i) the deficiency in the late stages of B lymphopoiesis in the two mutants is mostly ADAR1-dependent; and (ii) the more profound maturation block in the Adar^fl/fl^Cd19-Cre^ki/ki^ genotype is linked to an almost complete penetrance of *Adar* ablation in late B cell precursors of these mutant mice.

### ADAR1 is required for IL-7-dependent *in vitro* generation of late B cell precursors

**Figure 3:**
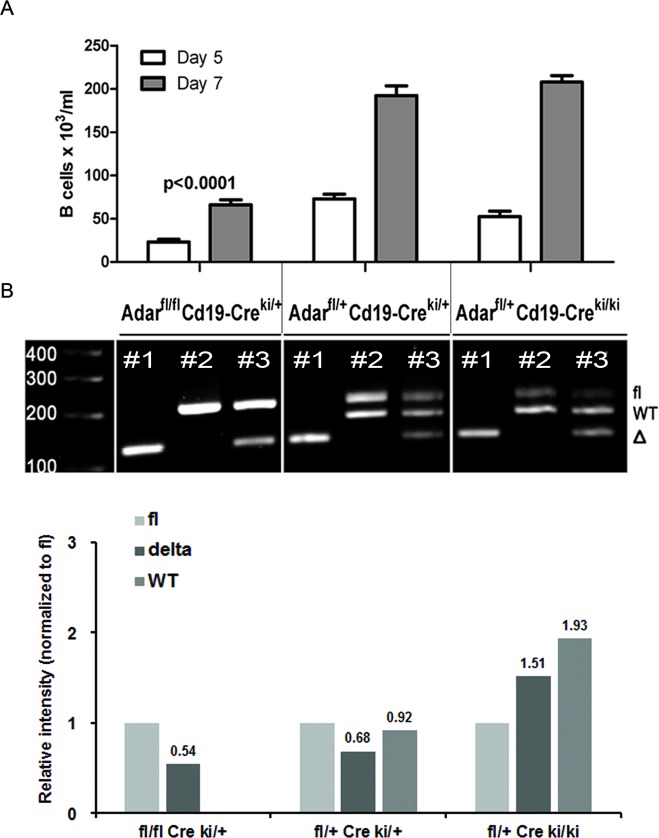
B cell precursors from Adar^**fl/fl**^Cd19-Cre^**ki/+**^ display a significantly reduced capacity for IL-7-dependent generation of CD19 B cells *in vitro* **A.** Bone marrow cells supplemented with rhIL-7 were cultured for 5 or 7 days, as described in methods. Cells were harvested, at the indicated time points, and absolute number of CD19^+^ B cells per mL was determined by FACS with the 123count eBeads kit. BM cultures were done in triplicates and bar graphs depict mean (±SD) from a representative experiment out of > 3 performed. Absolute CD19^+^ B cell counts in BM cultures of Adar^fl/fl^Cd19-Cre^ki/+^ mutants were significantly lower (*p* < 0.0001 by one-way ANOVA test) compared to the other groups at both time points. **B.** At the end of BM cultures (day 7), purified DNA from each respective sample was analyzed by PCR to detect relative quantity of either the *Adar* ∆ allele (lane 1), wt & fl alleles (lane 2), or all the three alleles simultaneously (lane 3). The image was acquired using a Molecular Imager ChemiDoc XRS^+^ system, and the densitometry analysis of the bands intensity was done using the Image Lab 5.1 software (BioRad Laboratories, Inc.).

To validate that the significant defect in late B cell precursor maturation in our model is mainly ADAR1-dependent, we cultured BM cells from Adar^fl/fl^Cd19-Cre^ki/+^ mutants (Cd19 sufficient) with IL-7 for 5 and 7 days. This *in vitro* culture system permitted us to study the expansion and maturation of IL7R^+^CD19^−^ B-lineage early precursors into B220^+^CD19^+^ late precursors (Fr. D/E). As predicted, we found that the *in vitro* generation of CD19^+^ B cell precursors was significantly reduced (*p* < 0.0001) at both time points in Adar^fl/fl^Cd19-Cre^ki/+^ mutants compared to control Adar^fl/+^Cd19-Cre^ki/+^ littermate cultures (Figure 3A). In contrast, *in vitro* generation of late B cell precursors was largely normal in Adar^fl/+^Cre^ki/ki^ BM cultures.

Additionally, to confirm ongoing Cd19-Cre-mediated *Adar* gene segment excision in our model, we analyzed the genomic DNA purified from day 5 cultures, by gel electrophoresis for the following PCR products: *Adar* ∆ allele alone (lane 1), wt & fl alleles (lane 2), or all the three alleles simultaneously (lane 3). We found that loxP site recombination was prominent *in vitro* in the three mice genotypes (Figure 3B, lane #1 in all samples). Moreover, the densitometry of band intensities of the Adar^fl/fl^Cd19-Cre^ki/+^ sample (lane #3), revealed that the relative level of ∆ allele was 0.54-fold compared to the *fl* allele (Figure 3B, see bar graph). This important finding demonstrates, as previously reported [10, 30], that incomplete penetrance of Cre-mediated *Adar* recombination results in selection against cells with biallelic *Adar* ablation. Moreover, Adar^fl/+^Cd19-Cre^ki/ki^ BM cultures displayed the highest efficiency of *Adar* recombination: the relative level of the ∆ allele (normalized to *fl* allele) was 1.51-fold compared to 0.68- and 0.54-fold in the other two littermate groups (Figure 3B, bar graph). Taken together these data confirm that Cd19-Cre^ki^ homozygosity is coupled with a several-fold higher efficiency of floxed gene segment recombination, supporting the conclusion that the substantial block in B lymphopoiesis in Adar^fl/fl^Cd19-Cre^ki/ki^ mice is mainly due to an almost complete penetrance of *Adar* ablation and its detrimental biological consequences.

### Adar ablation in B cell precursors leads to apoptosis and upregulation of typical ISGs

**Figure 4:**
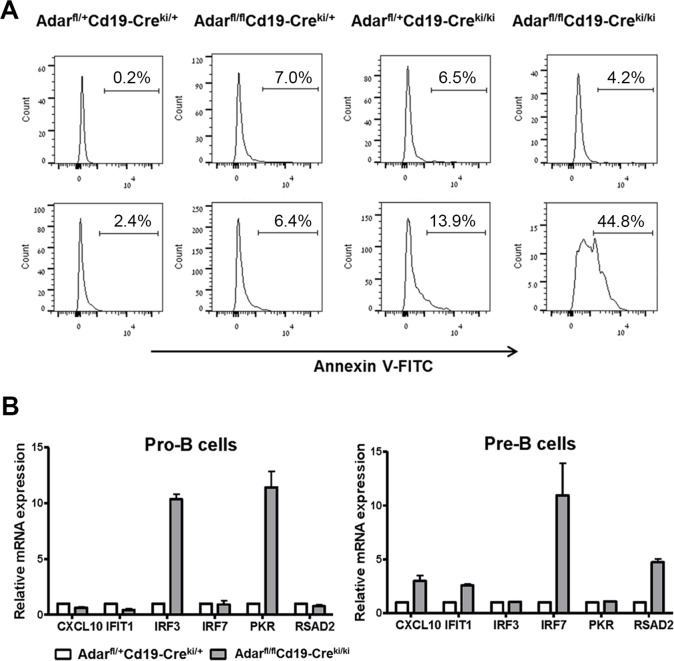
Cd19-Cre dependent disruption of Adar leads to apoptosis and type-I interferons response **A.** Histograms depict FACS analysis of Annexin V staining of pre-B cells (upper panel) and immature B cells (lower panel) following overnight *in vitro* incubation. **C.** Pro-B cells and pre-B cells were isolated from BMs by a FACSAria sorter, and total RNA was purified from the cells as detailed in Methods. Next, we measured by qRTPCR the relative mRNA expression of the indicated ISGs. Bar graphs depict mean of triplicates (± SD), as calculated by the ∆∆CT method, normalized to *Gapdh* gene expression. Data show a representative experiment out of three performed.

By Annexin-V staining of BM cells cultured overnight (Figure 4A), we found an increased rate of apoptosis of immature B cells from Adar^fl/fl^Cd19-Cre^ki/ki^ and Adar^fl/fl^Cd19-Cre^ki/+^ mutants compared to relevant littermate controls (~12- and ~4-fold increase, respectively). Our data agree with previous data showing that the loss of ADAR1 in Hematopoietic stem cells leads to rapid apoptosis [10]. The two major pathways previously linked to the induction of apoptosis in ADAR1 deficient cells include: (i) excessive activation of the dsRNA-dependent eukaryotic translation initiation factor 2-alpha kinase (Eif2ak2/PKR) with subsequent inhibition of protein synthesis; and (ii) dsRNA-dependent RIG-I-like receptors (RLR) mediated activation of IRF3 and a robust type-I interferons response [31, 32].

Next, to study whether *Adar* ablation in B cell precursors was linked to aberrant induction of the PKR and RLR dependent signaling pathways, we first isolated by FACSorting pro-B and pre-B cells from the BMs of Adar^fl/fl^Cd19-Cre^ki/ki^ mice and littermate controls. Total RNA from the FACS-sorted cells was then analyzed by qRTPCR for the relative quantity of a group of selected ISGs downstream of RLR activation (IRF7, CXCL10, RSAD2, and IFIT1). In pro-B cells, we found increased levels of IRF3 and PKR, both linked to the activation of pro-apoptotic pathways following ADAR1 silencing [33, 34]. In pre-B cells, we found increased transcription of all four selected ISGs (Figure 4B). Interestingly, IRF7 that was strongly upregulated in ADAR1 deficient pre-B cells is a major enhancer of the RLR mediated innate immune response [35, 36]; thus implying robust activation of this response in our model. Since the percentage of late B cell precursors (Fr. E/F) was extremely low in the BM of mutant mice, with the majority of these rare cells rapidly undergoing to apoptosis, we could not study the effect of *Adar* ablation directly in this relevantcell population.

Nucleic acids are the essential carriers of genetic information for all living organisms. Thus, selective identification of foreign nucleic acids is a major undertaking of the mammalian innate immune system [36]. Recent advances imply a role for dysregulation of nucleic acid sensing pathways in human autoimmunity [3, 13, 19]. In fact, mutations in ADAR1 are a genetic cause of the prototypic interferonopathy, AGS, displaying similarities with familial SLE including the production of distinct autoantibodies to nuclear proteins [14, 15]. Moreover, recent studies in mouse models show that genetic deletion of *Mavs* can rescue Adar^−/−^ mice to birth. Likewise, Adar^E861A/E861A^ knock in mice, expressing only a catalytically inactive ADAR1 and displaying embryonic lethality, could be rescued by concurrent knocking-out of *Ifih1*, encoding the cytoplasmic dsRNA sensor MDA5 [8, 9]. Activation of MDA5 induces the polymerization of MAVS that recruits and activates selected TRAF E3-ligases. This mediates the activation of the IKK and TBK1 kinases to phosphorylate IκBα and IRF3, respectively, leading to the robust transcription of ISGs and other cytokines [36, 37]. Taken as a whole these observations imply that a major biological function of ADAR1, conserved across mammalians, is the editing of repetitive sequences in transcripts that prevent the abnormal activation of MDA5 by self-dsRNAs. Indeed, our observation that a group of typical ISGs was upregulated in pre-B cells of Adar^fl/fl^Cd19-Cre^ki/ki^ mutant mice is consistent with the latter paradigm.

A previous report from the Nishikura laboratory [38] describes the use of Cd19-Cre mediated ablation of *Adar* to study its role in the biogenesis of miR-142 by splenic B cells. However, this report did not provide pertinent data regarding the effects of ADAR1 deficiency on B cell development and survival in the BM or spleen, and hence its relevance to our study is small. Pestal et al. have very recently reported that *Adar* and *Mavs* double knockout mice, surviving past 1 week of age, show a significant deficiency of mature peripheral B cells, but not of T cells and dendritic cells, within their peripheral lymphoid organs [17]. Unlike our work, the authors did not study the effect of germline *Adar* deletion on the process of B lymphopoiesis in the BM nor whether ADAR1 was vital, in this model, for the early (Fr. A/B), late (Fr. C-E) and/or final (Fr. F) B development. Importantly, our novel finding that ISGs are significantly upregulated in ADAR1 lacking pre-B cells is consistent with their observation, employing the Adar^p150−/−^ mouse model [39], that the B cell deficiency is ADAR1 p150 isoform dependent [17]. This interferon-inducible isoform, as opposed to the constitutive p110 isoform, performs its catalytic function mainly in the cytosol, playing a key role in the cytosolic regulatory network enabling tolerance to paired RNA components (dsRNAs) of the trascriptome [3, 19, 20].

Pertinently, we have just revealed that hepatocyte-specific ablation of ADAR1 induces massive liver damage with multifocal inflammation and hepatocyte apoptosis. The bioinformatics analysis of the whole transcript expression arrays from these abnormal livers indicated a robust upregulation of ISGs and related immune response genes [30]. Thus, we now communicate multiple data that *Adar*, is not only vital for stem/progenitor type cells in the fetal and early postnatal period, but also for more differentiated functional cells in adult mice: e.g., hepatocytes and newly formed immature B cells with functional surface IgM receptor.

In conclusion, we show for the first time that ADAR1, the master transcriptome editor, is essential for the late stages of B cell lymphoiesis in the BM and very likely for the survival of newly formed B cells in the periphery. Since the perturbation of nucleic acid sensing pathways and of autoreactive B cells plays a central role in SLE [40, 41] our data imply that further insight into the immunoregulatory role of ADAR1 may be relevant for a subset of autoimmune disorders.

## MATERIALS AND METHODS

### *In vivo* B cell-specific Cd19-Cre mediated conditional disruption of Adar

Mice with a loxP-flanked Adar1^f7-9^ (Adar^fl^) allele through the germline were previously described [11]. Homozygous Adar^fl/fl^ mice backcrossed into the C57BL/6 background were kindly provided by Stuart H. Orkin (Dana-Farber Cancer Institute, Boston, MA) and Peter H. Seeburg (Max Planck Institute for Medical Research, Heidelberg, Germany). The B6.129P2(C)-Cd19^tm1(cre)Cgn^/J deleter mice were obtained from The Jackson Laboratory (Bar Harbor, ME). The Cd19-Cre knock-in/knock-out allele (Cd19-Cre^ki^) has a *Cre* gene inserted into the first exon of the *Cd19* gene [27].

To generate mice with B cell lineage-specific disruption of *Adar* we first bred Adar^fl/fl^ mice to Cd19-Cre^ki/ki^ deleter mice to produce Adar^fl/+^Cd19-Cre^ki/+^offspring that were further interbred to generate four different genotypes. Two relevant mutant genotypes (Adar^fl/fl^Cd19^ki/ki^ / Adar^fl/fl^Cd19-Cre^ki/+^) with high and low efficiency of biallelic B cell-specific Adar^Δ7-9^ somatic gene segment excision, respectively, and two relevant littermate controls (Adar^fl/+^Cd19-Cre^ki/ki^ / Adar^fl/+^Cd19-Cre^ki/+^). Genomic DNA samples isolated from the tail tips of 3wk mice pups were analyzed by multiplex PCR that simultaneously detects Adar the wt(^+^), floxed(^fl^), and excised (^Δ7-9^) alleles, as previously described [11]. In paralleled, the presence of the Cd19-Cre^ki^ and/or the wt (^+^) allele was determined by a different multiplex PCR assay [27]. Additionally, to verify actual Cd19-Cre^ki^-dependent Adar gene disruption, selected DNA samples from BM cells of 8-16 week old mutant and littermate control mice were analyzed by multiplex PCR for the presence of *Adar* ^+^, ^fl^, or excised ^Δ7-9^ alleles. The mice were bred and housed in a SPF facility, and did not display any gross abnormalities as mature adults (3-6 months of age). The Institutional Animal Care and Use Committee of the Weizmann Institute of Science (Rehovot, Israel) approved all animal experiments.

### FACS analysis of B cell developmental stages

Single-cell suspensions of BM, Spleen, and of blood cells were obtained from 8-16 week old mice. Depletion of erythrocytes from the various sample was achieved using Red Blood Cell Lysis Buffer (Sigma-Aldrich Chemie GmbH, Steinheim, Germany). Next, the cells were immunostained with a relevant mixture of flourochrome conjugated mAbs against any of the following selected mouse B cell surface antigens B220/CD45R, CD19, CD43, HSA/CD24, CD25, AA4.1/CD93, CD23, IgM, and IgD; all obtained from eBioscience Inc. (San Diego, CA). Next, by polychromatic flow cytometry we analyzed the samples for relevant B cell developmental stages in the BM and periphery according to the Hardy classification and the Philadelphia nomenclature [26, 42]. The following B cells stages were identified: pro-B/Fr. B-C (B220^+^ CD25^−^ CD43^+^ CD24^++^ CD19^+/++^ CD93^+^); pre-B/Fr. D (B220^++^ CD25^+^ CD43^−^ CD24^++^ CD19^+++^ CD93^+^ IgM^−^); immature/Fr. E (B220^+++^ CD43+ CD24^hi^ CD19^+++^ CD93^+^ CD25^−^/IgM^+^ IgD^−^); mature recirculating/Fr. F (B220^+++^ CD19^+++^ CD23^++/hi^ CD93^−^); and splenic transitional T2 plus follicular (B220^+++^ CD19^+++^ CD23^++/hi^). Data were acquired on a FACSAria instrument (BD Biosciences, San Jose, CA), and analyzed/plotted using the FlowJo V.10 software (FlowJo, Ashland, OR).

### Isolation of pro- and pre-B cells from BM

Single-cell suspensions of BM derived cells from 8-16 week old mutant and littermate controls were depleted of red blood cells and immunostained for relevant cell surface markers, as detailed above. Subsequently, we sorted out pro-B cells and pre B-cells using a FACSAria instrument to high purity (~95%), as previously described [43]. Next, the purified cell were lysed with TRIzol^®^ Reagent (Invitrogen, Carlsbad, CA) and total RNA was purified using Direct-zol^™^ RNA Kits (Zymo Research Corporation, Irvine, CA) for subsequent downstream analysis. The quantification of selected gene transcripts was performed by quantitative PCR (qRTPCR) using specific predesigned pair of unlabeled PCR primers and a TaqMan^®^ probe (TaqMan^®^ Gene Expression Assays) and analyzed on an ABI PRISM 7300 Sequence Detection system (all reagents and the analyzer were from Applied Biosystems, Thermo Fisher Scientific Inc.). Selected DNA samples from pre-B cells of Adar^fl/fl^Cd19-Cre^ki/ki^ mutants and controls were also analyzed by multiplex PCR to verify enrichment for the Adar^Δ7-9^ allele in the mutant mice samples.

### BM cell cultures

To induce the IL7-dependent *in vitro* expansion and differentiation of early IL7R^+^ lymphoid progenitors, BM cultures were prepared as previously described [43]. Briefly, single cell suspension of BM cells obtained from the femurs of 8-16 week-old mice were first depleted of erythrocytes using Red Blood Cell Lysis Buffer (Sigma-Aldrich Chemie GmbH), per manufacturer's instructions. Then, BM cells were cultured, at 5 × 10^5^ cells/ml in a 6-well plate, for 5 and 7 days in Iscove modified Dulbecco medium supplemented with 100 IU/mL of recombinant human IL-7 (PeproTech, Rocky Hill, NJ). At the end of the experiment, we performed absolute counting of B220^+^ CD19^+^ cells by flow cytometry employing the 123count eBeads kit (eBioscience Inc.), according to manufacturer's instructions. As expected, usually at the end of culturing most of the cells were *in vitro* formed B220^int^CD19^+^ B cell precursors.

### Statistical analysis

Statistically significant differences between group means were determined by one-way ANOVA test with Dunnett's post hoc multiple comparison method, using Prism win V.5.02 (GraphPad Software, Inc. La Jolla, CA).
